# Transcriptome Profiling Reveals Differential Effect of Interleukin-17A Upon Influenza Virus Infection in Human Cells

**DOI:** 10.3389/fmicb.2019.02344

**Published:** 2019-10-10

**Authors:** Jing Li, Kun Zhang, Wenhui Fan, Shuang Zhang, Yun Li, Jinyan Gu, Jiyong Zhou, Wenjun Liu

**Affiliations:** ^1^CAS Key Laboratory of Pathogenic Microbiology and Immunology, Institute of Microbiology, Chinese Academy of Sciences, Beijing, China; ^2^Savaid Medical School, University of Chinese Academy of Sciences, Beijing, China; ^3^School of Dentistry, Philips Institute for Oral Health Research, Virginia Commonwealth University, Richmond, VA, United States; ^4^MOE Joint International Research Laboratory of Animal Immunology, Nanjing Agricultural University, Nanjing, China; ^5^MOA Key Laboratory of Animal Virology, Department of Veterinary Medicine, Zhejiang University, Hangzhou, China

**Keywords:** transcriptome analysis, H1N1 influenza virus, human cells, IL-17A, differential replication

## Abstract

Influenza A virus (IAV) has developed elegant strategies to utilize cellular proteins and pathways to promote replication and evade the host antiviral response. Identification of these sabotaged host factors could increase the number of potential antiviral drug targets. Here, IAV A/PR/8/34 (PR8)- and A/California/04/2009-infected A549 and 293T cells displayed differential virus replication. To determine the host cellular responses of A549 and 293T cells to IAV infection, RNA-seq was used to identify differentially expressed genes. Our data revealed that IAV-infected A549 cells activated stronger virus-sensing signals and highly expressed cytokines, which play significant roles in initiating the innate immune and inflammatory responses. In addition, IAV-infected 293T cells displayed weak immune signaling and cytokine production. Remarkably, IL-17A and associated genes were highly enriched in IAV-infected 293T cells. Furthermore, IL-17A can partially facilitate A549 cell infection by the PR8 strain and PR8-infected IL-17A knock-out mice consistently exhibited decreased weight loss and reduced lung immunopathology, as compared to controls. This work uncovered the differential responses of cells infected with two H1N1 IAV strains and the potential roles of IL-17A in modulating virus infection.

## Introduction

Influenza A virus (IAV) poses a substantial threat to human health in the forms of seasonal epidemics and occasional pandemics (Reperant et al., 2014). IAV mainly targets the epithelial cells of the respiratory tract, but can also infect macrophages and dendritic cells (Veckman et al., 2006). The innate immune system initiates host defenses against IAV infection, which is triggered by pattern recognition receptors (PRRs) that recognize viruses or their genetic material (Kawai and Akira, 2006; Wilkins and Gale, 2010). This recognition results in appropriate antiviral responses, including the production of a variety of cytokines and the induction of inflammatory and adaptive immune responses (Yoneyama and Fujita, 2010).

Dysregulation of the immune responses is correlated with increased morbidity and mortality due to IAV infection, including the highly pathogenic H5N1 virus, and the pandemic 2009 H1N1 IAV (Brandes et al., 2013; Teijaro et al., 2014). The overabundance of inflammatory cytokines is a major feature of 2009 H1N1 infection in both humans and experimental animals, and is widely thought to play a central role in the clinical outcome, and pathogenesis of IAV infection (Tisoncik et al., 2012; Hartshorn, 2013; Teijaro et al., 2014). Therefore, maintaining the immune system in an appropriately robust condition is thought to be important for the prevention of severe influenza symptoms (Yoneyama and Fujita, 2010; Iwasaki and Pillai, 2014; Teijaro et al., 2014; Netea et al., 2015). However, the molecular mechanisms underlying the balance between protective and pathological immune responses against IAV infection remain unclear.

Transcriptome analysis of host cellular responses to virus infection can be used to identify potential cellular factors directly or indirectly involved in viral infection (Huang et al., 2013). High-throughput RNA sequencing (RNA-seq) technology, a recently developed transcriptome profiling approach, combined with bioinformatics has emerged as an important tool to extract detailed information concerning cellular signaling mechanisms (Zou et al., 2013; Jones et al., 2014). RNA-seq technology has the potential to reveal dynamic alterations in the pathogen genome itself and the systemic changes in host gene expression profiles during the process of infection by pathogens, which could help clarify the pathogenesis of viral infection and interactive mechanisms of pathogens. RNA-seq has been applied to study various viral infections and diseases, although previous studies have primarily focused on influenza virus infection of mouse models (Xiao et al., 2013; Zou et al., 2013; Ertl and Klein, 2014; Jones et al., 2014; Wang et al., 2014). In addition, host transcript analyses have been conducted with the use of IAV-infected human lung tissues, which have confirmed significant enrichment of host defenses and the promotion of cell death, consistent with prior animal studies (Xiao et al., 2013).

In the present study, the abilities of mouse-adapted (A/PR/08/34) and non-adapted (A/CA/04/2009) H1N1 strains of IAV to induce differential defense responses in human cells were investigated. Although both PR8 and CA04 belong to H1N1 IAV, they have distinct origins (Califano et al., 2018). These two strains were used to infect human 293T cells as a representative human cell line and A549 cells (human lung epithelial cell line) as a more physiologically relevant cell line (Geiss et al., 2002; Temperton et al., 2007). RNA-seq was used to annotate host responses to infection of A549 and 293T cells by the PR8 and CA04 strains of H1N1 IAV. The results of this study provide a global view of virus type-specific and cell-specific mRNA profiles, illustrating the role, and mechanism of host/virus cellular responses during IAV infection. The differential infectivity of H1N1 IAV in A549 and 293T cells uncovered potential candidate genes that may contribute to the observed resilience of mammalian cells to pandemic IAV infection.

## Results

### H1N1 IAV Replicates Less Efficiently in 293T Cells

**FIGURE 1:**
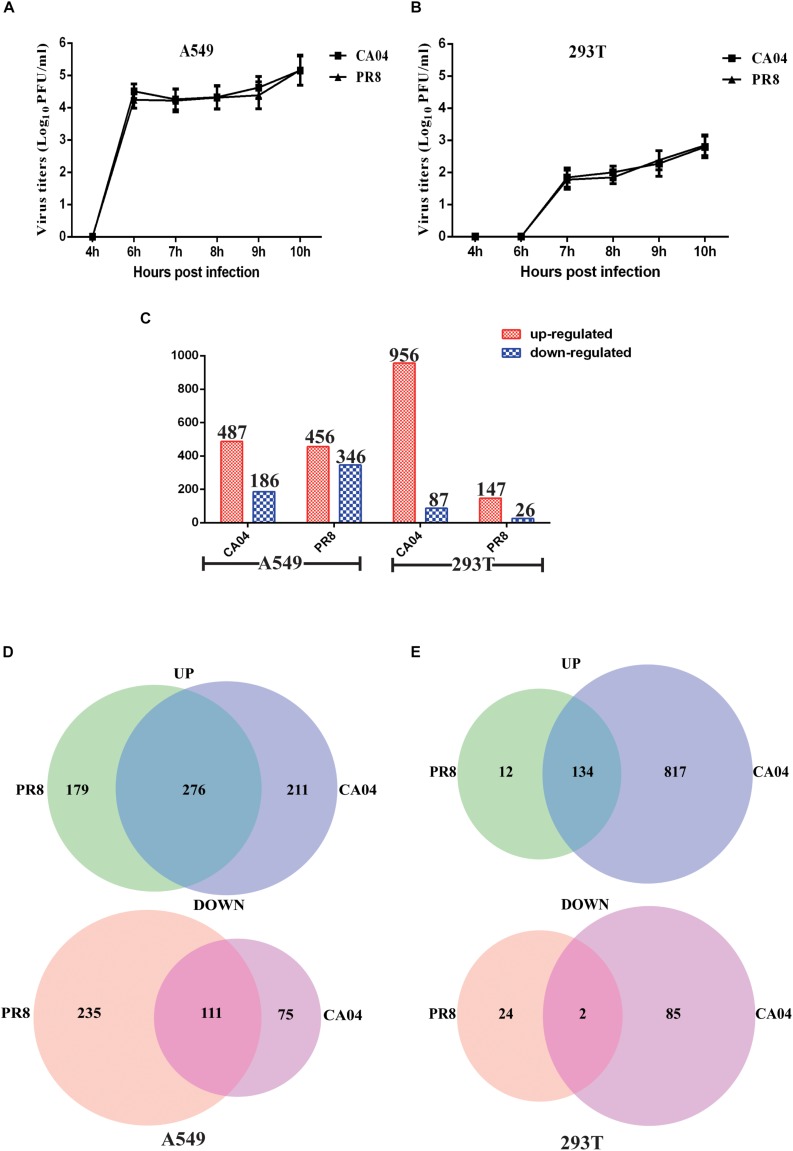
Viral titers and overview of the RNA-seq data of the DEGs. Differential replication efficiency and transcript profiles of responses to H1N1 IAV infection of A549 and 293T cells. **(A)** Growth characteristics of IAVs in A549 **(A)** and 293T **(B)** cells. The cells were infected with PR8 or CA04 virus (MOI = 1), respectively. Culture supernatants were collected at the indicated time points and viral titers were determined by plaque assays. **(C)** Numbers of reliably quantified DEGs (fold difference ≥2 or ≤0.5) in the A549 and 293T cells following infection with two different H1N1 IAV strains, as compared to uninfected controls. **(D)** Venn diagram showing the distribution of shared DEGs among PR8- and CA04-infected A549 **(D)** and 293T cells **(E)**.

To determine the efficiency of IAV replication in human cells, A549 and 293T cells were infected with CA04 and PR8 viruses (MOI = 0.1), respectively. Aliquots of cell supernatants were harvested at 4, 6, 7, 8, 9, and 10 h post-infection (hpi). The plaque assay was used to determine viral titers. Replication analysis in A549 cells revealed that propagation of PR8 and CA04 was similar in A549 cells over a 10-h period, reaching titers in the range of 5 log_10_ PFU/mL (Figure 1A). In contrast, there was a much lower viral titer increase for CA04 and PR8 in 293T cells (Figure 1B). The titers in 293T cells were > 200-fold lower than in A549 cells, indicating a lower replication efficiency of H1N1 IAV in 293T cells.

### Host Gene Response Profile

RNA isolates were prepared from both IAV- and mock-infected A549 and 293T cells at 8 hpi. A ≥2- or ≤0.5- fold difference in gene level and FDR-adjusted *p*-value < 0.05 between IAV-infected and mock infected extracts was considered as differential expression. As shown in Figure 1C, a total of 487 differentially expressed genes (DEGs) were up-regulated and 186 were down-regulated in A549 cells infected with CA04, while 456 DEGs were up-regulated and 346 were down-regulated in A549 cells infected with PR8. In addition, infection of 293T cells with strain PR8 resulted in fewer DEGs (147 up-regulated and 26 down-regulated). The Venn diagram shown in Figure 1D illustrates the overlap of A549 cell responses to CA04 and PR8 virus infection induced DEGs. 276 up-regulated and 111 down-regulated genes were DEGs in both virus infections. In contrast to A549 cells, the 293T cells infected with PR8 and CA04 virus demonstrated 134 common up-regulated genes but only two common down-regulated genes (Figure 1E). These results indicate differences between human cells in response to virus infection, with some shared alternations in expression profiles between the cell types.

### Comprehensive Analysis of Functional Enrichment in A549 or 293T Cells Infected With Two Different Viruses

To investigate possible biological interactions of DEGs and determine important functional networks, DEG datasets were imported into the Ingenuity pathway analysis (IPA) tool. To identify functional groups and components of processes that are common and unique between the responses of A549 and 293T cells infected with the two different H1N1 viruses, the statistical enrichment of canonical pathways identified by IPA (*p* < 0.01) was calculated. The 10 most common canonical pathways in infected A549 cells were interferon signaling and interferon signaling-associated pathways, indicating the rapid, and hyper-cytokine response in response to H1N1 IAV infection. In addition, the 10 most common pathways in infected 293T cells were involved in cytokine production regulation and various other pathways. Remarkably, the IL-17A and IL-17F pathways were highly enriched in IAV-infected 293T cells, indicating the potential function of IL-17 in the regulation of the IAV-induced inflammation responses of 293T cells (Table 1). To further explain the individual functional analyses, the top five canonical pathways of A549 or 293T cells infected by the two H1N1 IAV strains were identified (*p* < 0.01). Interestingly, the differential regulation of cytokine production in intestinal epithelial cells or macrophages by IL-17A and IL-17F were also enriched in the top five pathways in 293T cells infected with PR8 virus, further indicating the potential role of IL-17A/F in the regulation of virus propagation or the inflammatory responses of 293T cells.

**TABLE 1:** Canonical pathways enriched in common expressed genes (Top 10 enrichment).

**Infection models**	**Canonical pathway**	***p*-value**	**Mol^a^/mol^b^**
A549	Interferon signaling	3.88e-24	15/36
	Activation of IRF by cytosolic pattern recognition receptors	3.52e-13	11/64
	Role of pattern recognition receptors in recognition of bacteria and viruses	7.43e-10	11/127
	Antigen presentation pathway	3.92e-09	7/37
	Retinoic acid mediated apoptosis signaling	2.04e-07	7/64
	Role of RIG-I-like receptors in antiviral innate immunity	3.94e-07	6/49
	Communication between innate and adaptive immune cells	2.18e-06	7/112
	Role of JAK1, JAK2, and TYK2 in interferon signaling	1.66e-05	4/28
	Crosstalk between dendritic cells and natural killer cells	2.72e-05	6/106
	Type I diabetes mellitus signaling	7.59e-05	6/121
293T	Differential regulation of cytokine production in macrophages and T helper cells by IL-17A and IL-17F	1.04e-04	3/18
	Differential regulation of cytokine production in intestinal epithelial cells by IL-17A and IL-17F	2.22e-04	3/23
	Airway inflammation in asthma	2.63e-04	2/5
	Hepatic cholestasis	3.21e-04	6/177
	Agranulocyte adhesion and diapedesis	4.68e-04	6/190
	Granulocyte adhesion and diapedesis	2.41e-3	1/179
	Role of cytokines in mediating communication between immune cells	3.07e-03	3/56
	Activation of IRF by cytosolic pattern recognition receptors	4.47e-03	3/64
	T helper cell differentiation	6.22e-03	3/72
	TNFR2 signaling	9.85e-03	2/29

*IPA was used to determine the top 10 canonical pathways. The *p*-value was used to rank the significance associated with each pathway. ^*a*^Number of molecules differentially expressed in the canonical pathways. ^*b*^Total number of molecules in the annotated canonical pathways.*

**FIGURE 2:**
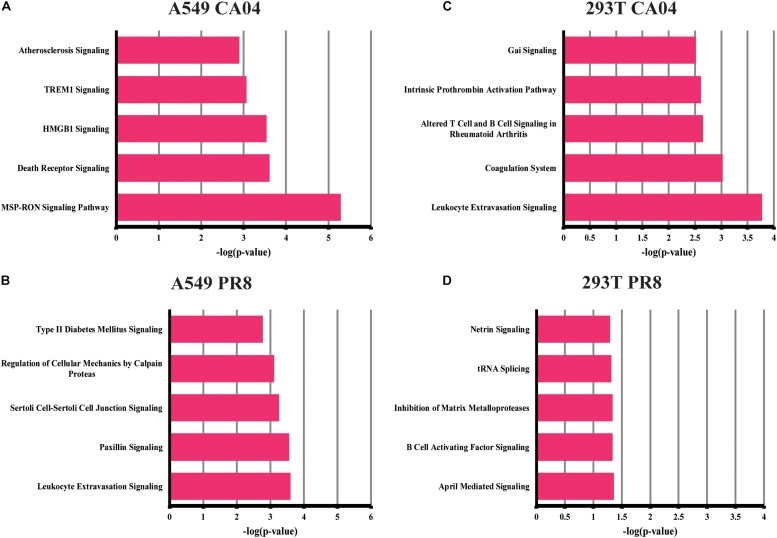
Top canonical signaling pathways of unique DEGs in CA04/PR8-infected A549 and 293T cells. Top five significant pathways associated with DEGs unique in CA04/PR8-infected A549 and 293T cells were enriched using IPA software (*p* < 0.01). **(A)** A549 cells infected with CA04 virus. **(B)** A549 cells infected with PR8 virus. **(C)** 293T cells infected with CA04 virus. **(D)** 293T cells infected with PR8 virus.

We also focused our attention on unique DEGs in CA04/PR8-infected cells. The top five pathways enriched in unique DEGs of CA04/PR8-infected A549 or 293T cells are displayed in Figure 2 (*p* < 0.01). Among these most significant regulated pathways, two of them are strongly associated with lymphocyte cell activation: MSP-RON signaling and HMGB1 signaling, which were only enriched by CA04 infected A549 cells. These results suggested that the CA04 strain may be able to disrupt lymphocyte function, thereby delaying efficient antiviral defenses by the host.

To examine the biological roles of the identified DEGs, functional analyses of both common and individual genes were performed using IPA software. Among the disease and disorder items, we focused on the inflammatory response, since many genes related to antiviral and inflammatory response signaling are included in this category (Supplementary Table S1). Interestingly, a greater number of genes were included in the H1N1 virus infected A549 cells, indicating a greater inflammatory response during infection in A549 cells. Furthermore, IPA upstream regulator analysis revealed that many of the top upstream regulators are associated with the type I IFN response in A549 cells, while IL17A was enriched in 293 T cells, demonstrating a potential function of IL17A during virus replication (Supplementary Table S2).

### Confirmation of DEGs by qRT-PCR

**FIGURE 3:**
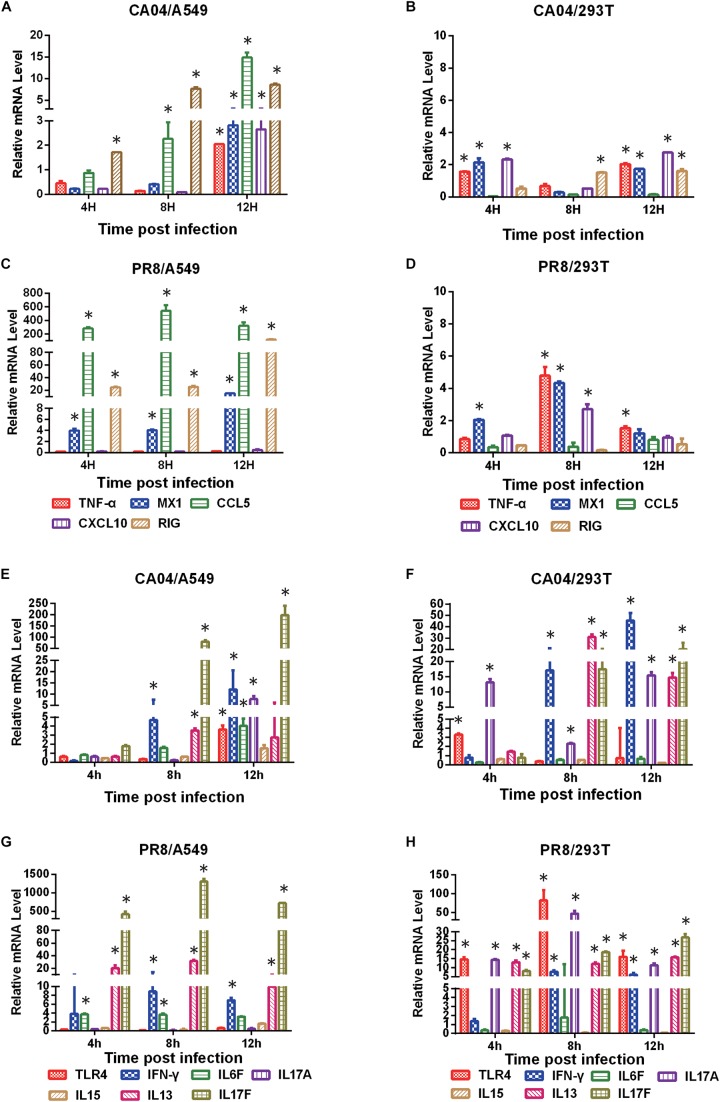
Verification of DEGs by qRT-PCR. The qRT-PCR results showed the same relative regulation of DEGs as the RNA-seq data. qRT-PCR analysis of the expression profiles of selected genes in CA04- and PR8-infected A549 or 293T cells relative to uninfected controls **(A, C, E G)**, A549 cells were infected with CA04 virus. **B, D, F, H**, 293T cells were infected with PR8 virus). The fold difference was determined using the 2^–DDCt^ method and RNA levels were normalized to β-actin. Data are presented as the mean ± standard deviation (SD) (*n* = 3, ^∗^*p* < 0.05).

To validate the differential expression profiles of genes obtained by RNA-seq analysis, quantitative RT-PCR was performed on selected DEGs. We found that the qRT-PCR results were consistent with those of the RNA-seq analysis. Although differences were observed between these two types of analyses due to intrinsic differences between the techniques, the qRT-PCR results displayed the same relative regulation of DE genes as the RNA-seq data (Figure 3). Consistent with virus propagation during the time course, most of the DEGs displayed higher mRNA levels in A549 cells than 293T cells, indicating the boosted cellular response in A549 cells upon influenza virus infection.

### Effect of IL-17A on Pathogenicity and the Regulation of Inflammatory Response Following H1N1 Virus Infection

**FIGURE 4:**
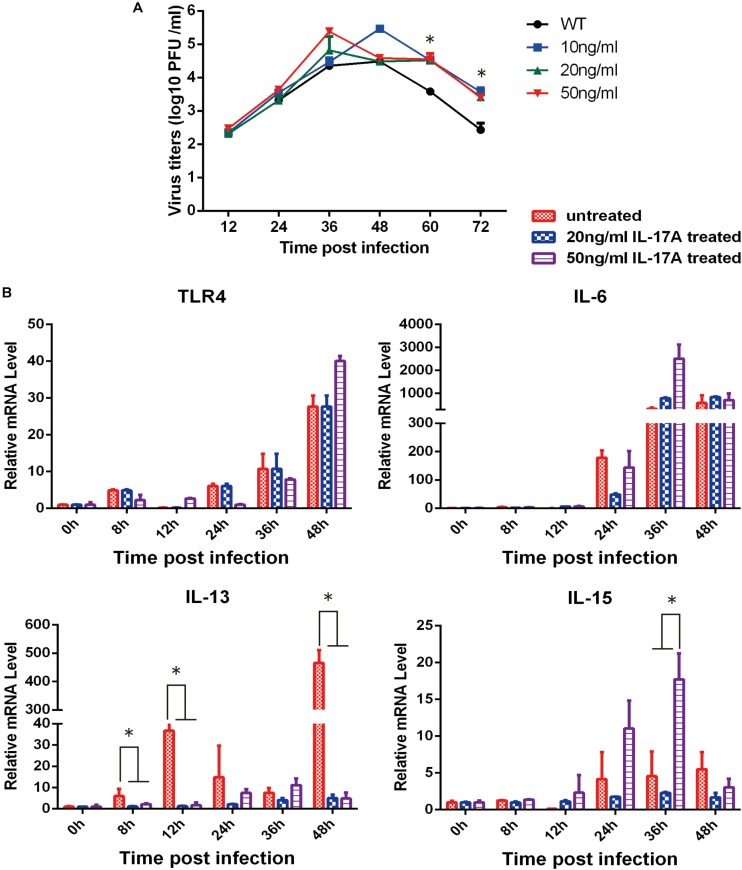
Functional analysis of IL-17A in PR8-infected A549 cells. The stimulation of IL-17A in A549 cells facilitated replication of the PR8 strain. **(A)** A549 cells were cultured with or without IL-17A (20 or 50 ng/mL) for 24 h and then infected with H1N1 IAV at a MOI of 0.01. Culture supernatants were collected at the indicated time points to determine viral titers by TCID_50_. **(B)** Cells were harvested at the indicated time points and cellular RNA was extracted to analyze a select panel of immune-related genes by qRT-PCR. RNA levels were normalized to β-actin. Fold differences were determined using the 2^–*DDCt*^ method as compared to uninfected cells. Data are presented as the mean ± SD (*n* = 3, ^∗^*p* < 0.05).

To further validate our approach for identifying functionally relevant host genes, A549 cells were treated with IL-17A protein and then infected with PR8 influenza virus. With increasing amounts of IL-17A protein, the viral titers also significantly increased at 60 and 72 hpi (Figure 4A). We next explored the impact of IL-17A stimulation on the expression of inflammatory response genes during IAV infection of A549 cells. IL-13, which is an anti-inflammatory factor during IAV infection. IL-17A stimulation resulted in marked down-regulation of IL-13 at all-time points post-infection when compared to untreated controls. In contrast, IL-15 expression was increased within higher concentrations IL-17A treatment (50 ng/mL) (Figure 4B).

**FIGURE 5:**
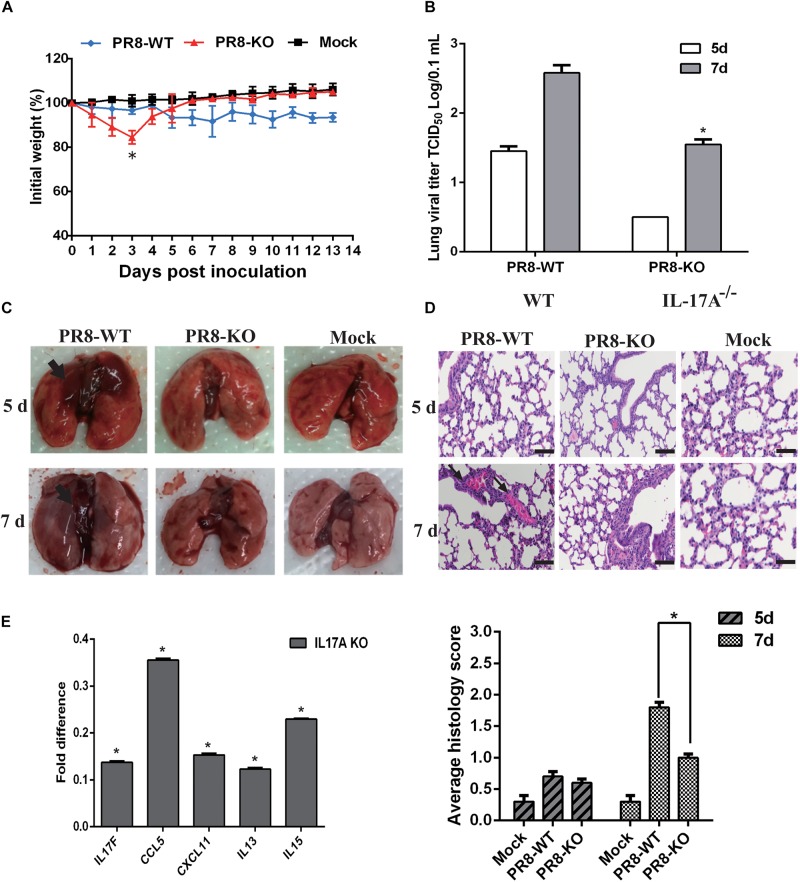
Functional analysis of IL-17A in a PR8-infected mouse model. The IL-17A knock-out mice displayed decreased virulence after infection with PR8 influenza virus. **(A)** IL-17A knock-out or WT mice were infected with PR8 influenza virus. Body weights were determined daily for 14 days and are depicted as the percentage of body weight at the time of inoculation. The data are the mean body weights of five mice. The error bars represent the standard error of the mean (*n* = 5, ^∗^*p* < 0.05). **(B)** Three mice from the PR8-infected groups were euthanized on days 5 and 7 post-infection for lung virus titration and necropsy examination **(C)**. **(D)** The lung tissues of PR8-infected IL-17A knock-out and WT mice were collected, formalin fixed, paraffin embedded, and stained with hematoxylin and eosin for histological analysis. Representative images are shown at 20× magnification. Mean histological scoring of lung sections were also measured (*n* = 3, ^∗^*p* < 0.05). **(E)** The relative levels of IL17F, CCL5, CXCL11, IL-13, and IL-15 in IL-17A knock-out mouse lungs were determined by qRT-PCR at 5 days after PR8 infection. RNA levels were normalized to β-actin. The fold differences from the qRT-PCR were determined using the 2^–Δ^ ^Δ^ ^Ct^ method and are expressed as fold induction compared to WT mice with PR8 infection. Data are presented as the mean ± SD (*n* = 3, ^∗^*p* < 0.05).

An IL-17A knock-out mouse model was employed to further determine the function of IL-17A during influenza virus infection, and we utilized an IL-17A knock-out mouse model. BALB/c IL-17A knock-out or wild-type (WT) mice were infected with 10^6^ TCID_50_ of PR8 influenza virus and then followed-up for 14 days to evaluate weight loss and survival. The PR8 virus elicited slightly decreased weight loss in IL-17A knock-out mice on early time points compared to WT animals. However, the KO mice recovered quickly and there were no deaths. Notably, none of the mice died during this experiment (Figure 5A). In contrast, CA04-infected WT mice displayed rapid weight loss when compared with IL-17A knock-out mice and slightly recovered within the evaluation period (Supplementary Figure S2). Since IAV primarily infects the mouse lung, lung samples were collected from infected animals to determine the viral titers at the indicated time points. Interestingly, as compared to the WT mice, H1N1 titers were significantly decreased in the lung tissues of IL-17A knock-out mice at 5 and 7 dpi (Figure 5B). Similar results were obtained in IL-17A knock-out mice infected with the CA04 strain (Supplementary Figure S2). Furthermore, necropsy examinations also indicated significantly alleviated pneumonia lesions at 5 and 7 dpi in IL-17A knock-out mice infected with PR8 virus (Figure 5C). Lung samples were also harvested from IL-17A knock-out and WT mice following infection with the PR8 virus to assess histological changes resulting from infection. The WT mice displayed slight pulmonary damage after infection with PR8 influenza virus at 7 dpi, presenting with moderate thickening of the alveolar walls and prominent infiltration of neutrophils, lymphocytes, and macrophages (Figure 5D). In addition, levels of inflammatory cytokines and chemokines in lung homogenates were measured at 5 dpi. Interestingly, the expression levels of all genes were dramatically decreased in the IL-17A knock-out mice, as compared to the WT mice, after PR8 infection. Importantly, there was also a significant decrease in IL-17F (Figure 5E).

## Discussion

Host defense barriers that prevent transmission of influenza viruses from one species of animal to another do not allow the virus to grow efficiently in other species. The receptor specificity of the hemagglutinin and internal genes of influenza viruses play roles in host range restriction (Kuiken et al., 2006). In addition to the virus itself, host restriction proteins also play important roles in virus replication (Brass et al., 2009). Although numerous studies have documented the roles of various influenza virus proteins in immune evasion, factors that maintain the balance between protective and pathological host responses to infection are less well understood (Gao et al., 2009; Wu et al., 2011; Zhang et al., 2012; Taubenberger and Morens, 2013). The application of genome-wide profiling techniques, such as microarray analysis, has resulted in the identification of novel host factors involved in H1N1 IAV infection of mice, ferrets, and macaques (Cameron et al., 2008; Boon et al., 2009; Cilloniz et al., 2010; Go et al., 2012; Shinya et al., 2012). In addition to animal models, RNA-seq technology is also widely used to explore host cell responses to human IAV infection (Chakrabarti et al., 2010; Fabozzi et al., 2018; Russell et al., 2018). In this experiment, two different H1N1 IAV strains were used to independently infect A549 and 293T cells. RNA-seq was used to identify host DEGs during infection with two different H1N1 IAV strains. Numerous genes displayed significantly altered expression levels upon infection with different virus types. To better understand the host cell response to H1N1 IAV infection at 8 h, a total of 249 common DEGs were identified in A549 cells infected with the two different strains, but only 137 common DEGs were identified in CA04- and PR8-infected 293T cells, corresponding to the lower viral titers in 293T cells. Our previous work demonstrated that DEGs in H5N1-infected 293T and A549 cells were mainly linked to cellular function and metabolic processes (Cao et al., 2019). These differential expression patterns motivated us to investigate the complicated relationship between H1N1 IAV and host cells.

Intense inflammatory responses are consistently reported as features of the high pathogenesis in humans (Jin et al., 2014). Using common genes and total genes analysis, IPA data analysis (canonical pathways and functional analysis) revealed that the major host response pathways for A549 cells upon infection with PR8 or CA04 virus were PRR and interferon-related pathways. Similar results were also observed in a previous study, most up-regulated genes in PR8 infected A549 cell were also significantly enriched in the innate immunity, type I interferon response and PRR pathways (Zhou et al., 2017). These results demonstrated that A549 cells infected with the H1N1 virus induced robust IFN responses and the activation of interferon-stimulated genes at early time points. In contrast to a prior report (Zhou et al., 2017), other pathways involved in leukocyte extravasation signaling or paxillin signaling were also enriched in unique DGEs of A549 cells infected with PR8 virus, indicating that the potential functional genes were hiding behind the robust interferon response and antiviral response upon influenza virus infection. In addition, H1N1 IAV-infected 293T cells apparently failed to initiate the cellular inflammatory response and immune response. In addition, IL-17A- and Th17-associated pathways were highly enriched in 293T cells upon H1N1 IAV infection, indicating that 293T cells are not very prone to triggering host anti-influenza viral responses, although the cell is usually used for packaging virus (Gao et al., 2009; Russell et al., 2018).

In CA04 virus-infected A549 cells, specifically enriched pathways were strongly associated with lymphocyte functions: HMGB1 signaling and MSP-RON signaling pathways. HMGB1 is an alarmin, which functions as endogenous molecular to activate the innate immune response (Klune et al., 2008). The MSP-RON pathway also plays an import role in the activation of macrophages and natural killer cells (Wilson et al., 2008). A previous study reported H5N1 virus PB1-F2 inhibited host lymphocyte cell activation through inhibiting HMGB1 and MSP-RON pathways (Leymarie et al., 2013). These pathways may also be utilized in CA04-virus infected A549 cells to subvert host defense responses. Although similar virus replication was displayed in CA04/PR8 was displayed in infected A549 cells, completely different enriched pathways indicated the differential host response evasion strategies of H1N1 IAV.

IL-17, which is produced during the adaptive and innate immune responses, functions as a pro-cytokine and plays a critical role during H5N1 IAV infection (Wang et al., 2011; Cypowyj et al., 2012). A recent study also demonstrated a protective role of IL-17A by modulating early humoral immunity (Wang et al., 2016). However, previous studies of IL-17RA-deficient mice reported that IL-17 may act as a double-edged sword by contributing to pulmonary immunopathology in pandemic H1N1 virus infection (Li et al., 2012; Xue et al., 2017). Indeed, the H5N1-infected IL-17-deficient subjects display increased weight loss and reduced survival rates (Wang et al., 2011). However, the functions of IL-17A during IAV infection still need to be elucidated *in vitro* and *in vivo*.

In the present study, stimulation of A549 cells by IL-17A partially enhanced replication of PR8 virus. IL-17A can promote the release of IL-15, and most interestingly, can significantly suppress IL-13 expression. Previous reports indicate that IL-13 production leads to impaired antiviral immune responses, resulting in a more severe influenza virus infection (Chang et al., 2011). We speculate that IL-17A stimulation during H1N1 infection in A549 cells may limit the inflammatory response by regulating IL-13 expression. Consistent with previous research, we observed that IL-17A knock-out mice achieved higher survival rates and the lungs of PR8-infected IL-17A knock-out mice had lower viral titers than those of WT animals. Tissue-specific micro-environmental factors may respond to these differential reactions *in vivo* and *in vitro*. Nonetheless, further studies are needed to explore the mechanisms underlying the influence of cellular components on lung pathology and the inflammatory response during influenza virus infection in IL-17A knock-out mice.

## Materials and Methods

### Ethics Statement

The mouse experimental design and protocols used in this study were approved by “the regulation of the Institute of Microbiology, Chinese Academy of Sciences of Research Ethics Committee” (Permit Number: PZIMCAS2013001). All mouse experimental procedures were performed in accordance with the Regulations for the Administration of Affairs Concerning Experimental Animals approved by the State Council of the People’s Republic of China. Studies with all H1N1 AIVs were conducted in a biosecurity level 2 laboratory.

### Reagents, Cell Lines, and Virus Preparation

Recombinant human interleukin (IL)-17A and IL17F were purchased from R&D Systems, Inc (Minneapolis, MN, United States). Human embryonic kidney cells (293T), Madin-Darby canine kidney (MDCK) epithelial cells, and human lung carcinoma (A549) epithelial cells were grown in Dulbecco’s modified Eagle’s medium (DMEM, Gibco) supplemented with 10% fetal bovine serum (FBS, Gibco) at 37°C in 5% CO_2_. All cells were purchased from ATCC.

The A/H1N1/Puerto Rico/8/34 (PR8) and A/California/04/2009 (CA04) strains were stored in our laboratory. All viruses were grown in MDCK cells for 72 h at 37°C. Viral titers were determined by plaque assays.

### Virus Infection and RNA Preparation

For virus infection, 293T or A549 cells were incubated with H1N1 IAV (PR8 or CA04) at a MOI of 0.1 for 1 h at 37°C. The cells were then washed with phosphate-buffered saline (PBS) and incubated in DMEM with or without FBS for the indicated times at 37°C in 5% CO_2_. The infected cells were collected and used for RNA extraction. Total RNA was extracted using the RNeasy Mini kit (Qiagen). RNA quality was assessed by analysis of the rRNA band integrity.

### cDNA Library Preparation and Next Generation Sequencing

For cDNA library preparation, total RNA was treated with RNase-free DNase I (TaKaRa) according to the manufacturer’s instructions. Then, the RNAs were quantified using a NanoDrop ND1000 (Thermo-Fisher Scientific, Waltham, MA, United States), and their quality was assessed with an Agilent 2100 Bioanalyzer (Agilent, Santa Clara, CA, United States). The RNA integrity number (RIN) of each sample was > 8. The cDNA libraries were prepared according to the standard Illumina protocol. The RNA-seq was performed by the Illumina HiSeq 4000 platform to generate 150-bp paired-end reads. The three sets (repeat 3 times for each group) of different cell samples were used. RNA-seq raw data were cleaned by removing the adaptor sequence and low-quality reads (Supplementary Table S4). To annotate and evaluate the transcript abundances from the sequenced reads, the human genome (hg19) and annotation for protein-coding genes were downloaded from the University of California Santa Cruz (UCSC)^1^ and served as references. After filtering reads containing sequencing adapters and reads of low quality, the remaining reads were aligned to the human genome using Tophat and normalized expression were calculated using the RPKM method. The distributions of reads for known genes were analyzed using the HTSeq. All Seq genes expressed in at least one of the samples with RPKM ≥ 0.1 were used for the analysis. RNA-seq raw data are included as Supplementary Material.

### Transcriptional Profiling Analysis

Ingenuity pathway analysis software (Ingenuity System) was used for DEG analysis, and the false discovery rate (FDR)-adjusted *p* < 0.05 was considered to indicate DEGs. Genes with ≥ 2-fold changes were selected for further analysis. Gene ontology (GO) functional classifications were determined using Blast2GO software, and the enriched gene functional categories were further classified by GO analysis with *p* < 0.05. Data were analyzed using IPA software. The most significant canonical pathways and functional processes of biological importance were identified using the list of DEGs identified by RNA-seq. Upstream regulator analysis was used to predict regulators and infer their activation state according to prior knowledge of expected effects and known target genes from the IPA database.

### Host Gene Expression Analysis by qRT-PCR

Total RNA was extracted from IAV-infected or mock-infected cells using the RNeasy Mini kit. RNA was reverse transcribed into cDNA using a Superscript II reverse transcriptase kit (Invitrogen). qPCR was performed using Brilliant SYBR Green qPCR master mix (Agilent Technology) with specific primers. The relative expression of mRNA was normalized to β-actin expression. Primers are listed in Supplementary Table S3.

### *In vivo* Infections

Female BALB/c mice (6- to 8-weeks-old) and IL-17A knock-out BALB/c mice were intranasally infected with 10, 10^3^, or 10^5^ TCID_50_ of PR8 virus or PBS control. The body weight was monitored daily. On days 5 and 7 post-infection, the mice were euthanized under anesthesia and necropsies were performed. Tissue samples from the lungs were homogenized in PBS supplemented with antibiotics to determine viral titers by TCID_50_.

### Histopathology Analysis

Lung tissues were fixed in 10% neutralized phosphate-buffered formalin. Fixed tissues were dehydrated, embedded in paraffin, and cut into 5 μm-thick sections, which were stained with hematoxylin and eosin. Histopathological lesions were measured on the basis of neutrophils, lymphocytes and macrophages cell infiltrations (0, none-3, extreme).

### Statistical Analyses

Ingenuity pathway analysis software (Ingenuity System) was used for DEG analysis, and the false discovery rate (FDR)-adjusted *p* < 0.05 was considered to indicate DEGs. Statistically significant differences between experimental groups were evaluated using the Student’s *t*-test and analysis of variance (ANOVA) with GraphPad Prism 6 software (GraphPad Software, Inc., La Jolla, CA, United States). *p* < 0.05 was considered statistically significant.

## Data Availability Statement

All datasets generated for this study are included in the manuscript/Supplementary Files.

## Ethics Statement

The mouse experimental design and protocols used in this study were approved by “the regulation of the Institute of Microbiology, Chinese Academy of Sciences of Research Ethics Committee” (Permit Number: PZIMCAS2013001). All mouse experimental procedures were performed in accordance with the Regulations for the Administration of Affairs Concerning Experimental Animals approved by the State Council of the People’s Republic of China.

## Author Contributions

JL, WL, and JZ conceived and designed the experiments. JL, WF, SZ, and YL performed the viral replication ability tests, animal experiments, histopathology, and immunology analyses. JL and KZ performed the RNA-seq and other experimental data analyses, prepared the manuscript, and completed its revision. JG suggested many of the experiments in this study. All authors read and approved the final manuscript.

## Conflict of Interest

The authors declare that the research was conducted in the absence of any commercial or financial relationships that could be construed as a potential conflict of interest.

## Footnotes

1 http://genome.ucsc.edu

## Supplementary Material

FIGURE S1 Top five canonical signaling pathways of total DEGs in CA04/PR8-infected A549 and 293T cells. **(A)** A549 cells infected with CA04 virus. **(B)** A549 cells infected with PR8 virus. **(C)** 293T cells infected with CA04 virus. **(D)** 293T cells infected with PR8 virus.

FIGURE S2 Functional analysis of IL-17A in a CA04-infected mouse model. **(A)** IL-17A knock-out or WT mice were infected with CA04 influenza virus. Body weights were determined daily for 14 days and are depicted as the percentage of body weight at the time of inoculation. The data are the mean body weights of five mice; the error bars represent the standard error of the mean (*n* = 5, ^∗^*p* < 0.05). **(B)** Three mice from the CA04-infected groups were euthanized on days 5 and 7 post-infection for lung virus titration examination (*n* = 3, ^∗^*p* < 0.05).

TABLE S1 Functional analysis of common DEGs from A549 and 293T cells infected with CA04 or PR8 virus.

TABLE S2 Upstream regulator analysis of common DEGs from A549 and 293T cells infected with CA04 or PR8 virus.

TABLE S3 Primers use in this study.

TABLE S4 Summary of RNA-seq data.

TABLE S5 RNA-seq data for CA04 virus infected 293T cells.

TABLE S6 RNA-seq data for PR8 virus infected 293T cells.

TABLE S7 RNA-seq data for CA04 virus infected A549 cells.

TABLE S8 RNA-seq data for PR8 virus infected A549 cells.
