# Supplementary figures and images for: Transcriptome Profiling Reveals Differential Effect of Interleukin-17A Upon Influenza Virus Infection in Human Cells
Source: Front Microbiol. 2019 Oct 10;10:2344. doi: 10.3389/fmicb.2019.02344 (PMC6798183; doi:10.3389/fmicb.2019.02344)

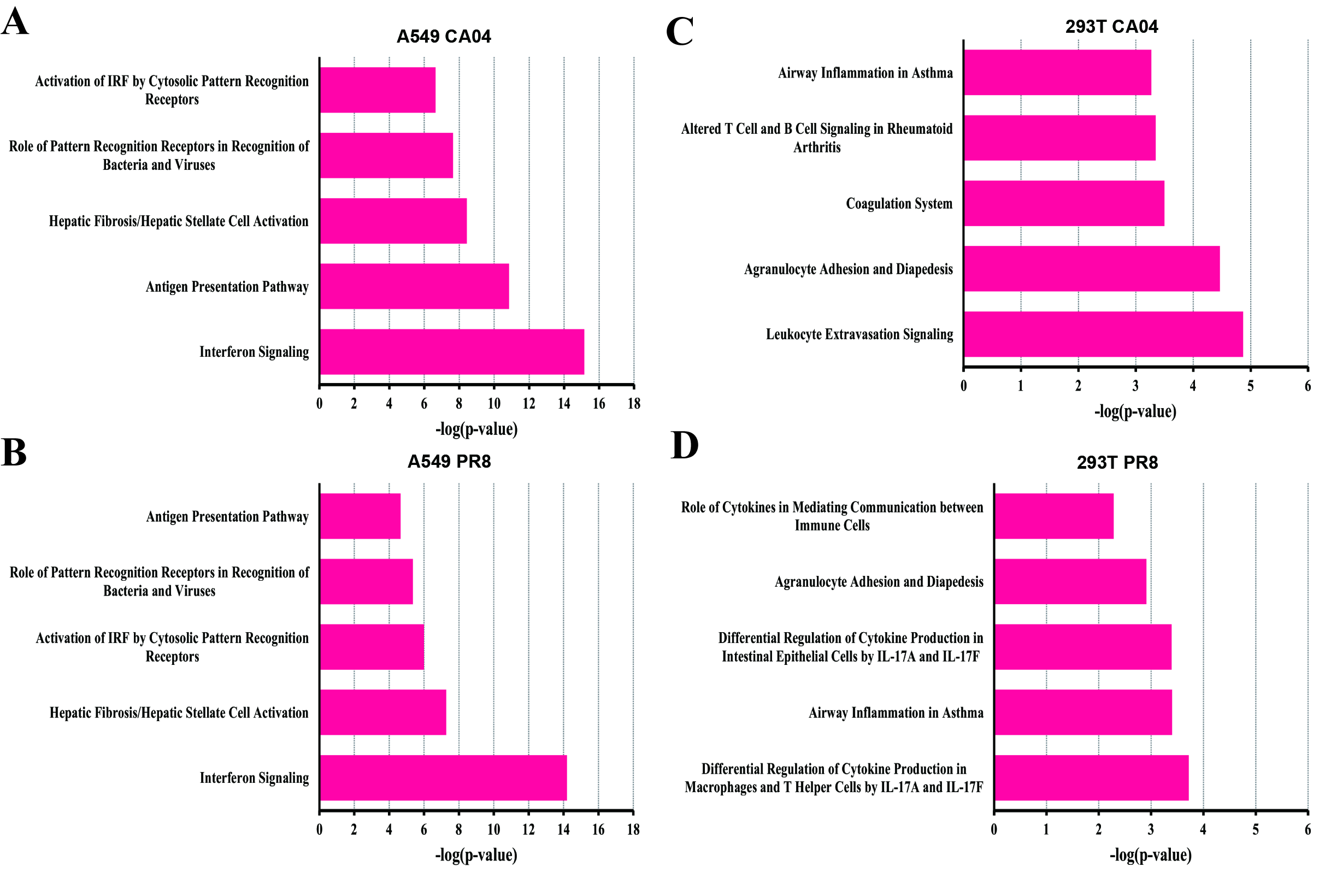

Supplement: FIGURE S1 — Top five canonical signaling pathways of total DEGs in CA04/PR8-infected A549 and 293T cells. (A) A549 cells infected with CA04 virus. (B) A549 cells infected with PR8 virus. (C) 293T cells infected with CA04 virus. (D) 293T cells infected with PR8 virus. [file Image_1.TIF]

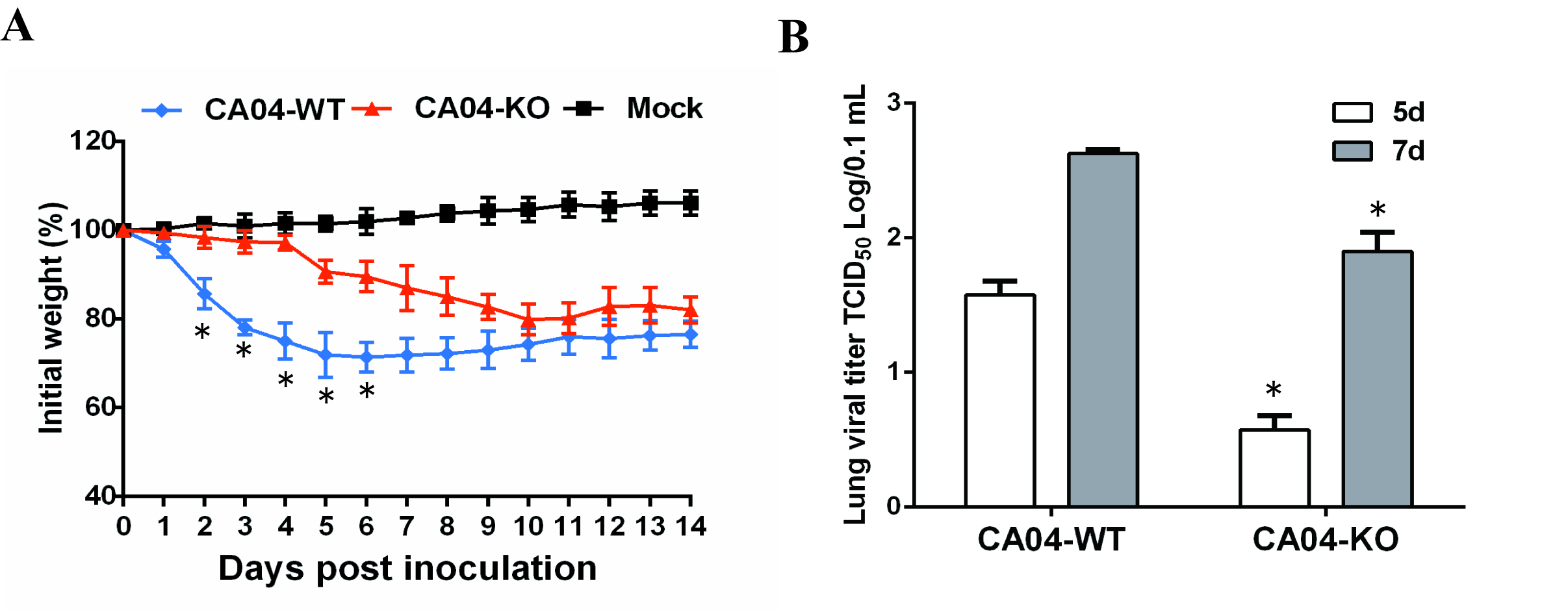

Supplement: FIGURE S2 — Functional analysis of IL-17A in a CA04-infected mouse model. (A) IL-17A knock-out or WT mice were infected with CA04 influenza virus. Body weights were determined daily for 14 days and are depicted as the percentage of body weight at the time of inoculation. The data are the mean body weights of five mice; the error bars represent the standard error of the mean (n = 5, ∗p < 0.05). (B) Three mice from the CA04-infected groups were euthanized on days 5 and 7 post-infection for lung virus titration examination (n = 3, ∗p < 0.05). [file Image_2.TIF]
