# Supplementary material for: Transcriptome Profiling Reveals Differential Effect of Interleukin-17A Upon Influenza Virus Infection in Human Cells
Source: Front Microbiol. 2019 Oct 10;10:2344. doi: 10.3389/fmicb.2019.02344 (PMC6798183; doi:10.3389/fmicb.2019.02344)
Supplement: TABLE S1 — Functional analysis of common DEGs from A549 and 293T cells infected with CA04 or PR8 virus. [file Table_1.docx]

**Supplement Table 1. Functional analysis of common DE genes from A549 and 293T cells infected with CA04 and PR8 virus**

| **Cell** | **Categories and Bio Functions** | ***p-*value range** | **No. Molecules** |
| --- | --- | --- | --- |
| ***A549*** | **Disease and Disorders** |  |  |
|  | Infectious disease | 5.41E-35 - 1.50E-03 | 65 |
|  | Antimicrobial Response | 1.34E-28 - 4.23E-04 | 26 |
|  | Inflammatory Response | 1.34E-28 - 1.43E-03 | 64 |
|  | Dermatological Diseases and Conditions | 2.37E-24 - 5.82E-04 | 45 |
|  | Immunological Disease | 9.19E-20 - 8.95E-04 | 60 |
|  | **Molecular and Cellular Functions** |  |  |
|  | Cellular Function and Maintenance | 5.24E-15 – 1.43E-03 | 39 |
|  | Cellular Development | 2.06E-14 - 1.50E-03 | 55 |
|  | Cell Death and Survival | 1.87E-12 - 1.50E-03 | 61 |
|  | Cell Signaling | 3.95E-12 - 1.17E-03 | 25 |
|  | Cellular Growth and Proliferation | 3.85E-11 - 1.50E-03 | 65 |
|  | **Physiological System Development and Function** |  |  |
|  | Hematopoiesis | 2.06E-14 - 1.50E-03 | 35 |
|  | Hematological System Development and Function | 2.40E-12 - 1.50E-03 | 50 |
|  | Organismal Survival | 3.41E-12 – 6.54E-07 | 30 |
|  | Tissue Morphology | 2.09E-11 - 1.30E-03 | 36 |
|  | Lymphoid Tissue Structure and Development | 3.60E-10 - 1.50E-03 | 26 |
|  |  |  |  |
| ***293T*** | **Disease and Disorders** |  |  |
|  | Cancer | 1.87E-05 - 1.03E-02 | 85 |
|  | Inflammatory Disease | 4.38E-05 - 1.03E-02 | 21 |
|  | Organismal Injury and Abnormalities | 5.94E-05 - 1.03E-02 | 34 |
|  | Respiratory Disease | 5.94E-05 - 1.03E-02 | 5 |
|  | Inflammatory Response | 1.04E-04 – 1.03E-02 | 22 |
|  | **Molecular and Cellular Functions** |  |  |
|  | Cellular Movement | 1.99E-05 - 1.03E-02 | 27 |
|  | Cellular Development | 7.94E-05 - 1.03E-03 | 15 |
|  | Cellular Function and Maintenance | 7.94E-05 - 1.03E-02 | 9 |
|  | Cellular Morphology | 1.04E-04 - 1.03E-02 | 10 |
|  | Cellular Growth and Proliferation | 1.57E-04 - 1.03E-02 | 14 |
|  | **Physiological System Development and Function** |  |  |
|  | Hematological System Development and Function | 1.99E-05 - 1.03E-02 | 21 |
|  | Immune Cell Trafficking | 1.99E-05 - 1.03E-02 | 17 |
|  | Cell-mediated Immune Response | 2.66E-05 - 5.98E-03 | 7 |
|  | Organismal Development | 5.94E-05 - 1.03E-02 | 14 |
|  | Cardiovascular System Development and Function | 7.20E-05 - 1.03E-02 | 17 |
|  |  |  |  |

Ingenuity Pathway Analysis was used to determine the top Bio Functions associated with the IPA Categories Disease and Disorders, Molecular and Cellular Functions, and Physiological System Development and Function. Fisher’s Exact test p-value was used to rank the significance associated for each Bio Function.
