# Supplementary material for: Transcriptome Profiling Reveals Differential Effect of Interleukin-17A Upon Influenza Virus Infection in Human Cells
Source: Front Microbiol. 2019 Oct 10;10:2344. doi: 10.3389/fmicb.2019.02344 (PMC6798183; doi:10.3389/fmicb.2019.02344)
Supplement: TABLE S4 — Summary of RNA-seq data. [file Table_4.DOCX]

**Supplement Table 4. Summary of RNA-Seq data**

| Sample | Raw reads | Clean reads | Clean bases | Error rate (%) | Q20 (%) | Q30 (%) | GC content (%) |
| --- | --- | --- | --- | --- | --- | --- | --- |
| CA04-A549 | 28,945,097 | 27,854,385 | 4.26G | 0.02 | 96.78 | 89.69 | 52.45 |
| CA04-A549 | 29,859,385 | 28,848,637 | 4.17G | 0.01 | 95.52 | 89.42 | 52.53 |
| CA04-A549 | 28,045,738 | 27,716,588 | 4.30G | 0.02 | 97.06 | 91.23 | 52.55 |
| CA04-293T | 28,987,256 | 28,012,496 | 3.92G | 0.02 | 96.12 | 91.47 | 52.12 |
| CA04-293T | 29,482,345 | 28,782,385 | 4.05G | 0.01 | 96.24 | 90.87 | 52.48 |
| CA04-293T | 28,855,081 | 27,714,024 | 3.97G | 0.02 | 95.43 | 89.84 | 52.32 |
| PR8-A549 | 29,947,516 | 28,827,592 | 4.15G | 0.01 | 96.88 | 90.93 | 52.27 |
| PR8-A549 | 29,594,852 | 28,241,540 | 4.21G | 0.02 | 96.49 | 89.14 | 52.38 |
| PR8-A549 | 29,094,068 | 28,194,812 | 4.25G | 0.01 | 96.82 | 90.22 | 52.26 |
| PR8-293T | 27,396,898 | 26,406,945 | 3.72G | 0.01 | 95.43 | 89.14 | 52.61 |
| PR8-293T | 28,048,570 | 26,958,992 | 3.82G | 0.02 | 95.17 | 89.21 | 52.57 |
| PR8-293T | 28,195,329 | 26,978,303 | 3.71G | 0.01 | 95.73 | 88.89 | 52.41 |
